# Influence of Chloride-Ion Adsorption Agent on Chloride Ions in Concrete and Mortar

**DOI:** 10.3390/ma7053415

**Published:** 2014-04-30

**Authors:** Gai-Fei Peng, Nai-Qian Feng, Qi-Ming Song

**Affiliations:** 1 Faculty of Civil Engineering, Beijing Jiaotong University, Beijing 100044, China; E-Mail: jiancaiyanjiu@163.com; 2 Department of Civil Engineering, Tsinghua University, Beijing 100084, China; E-Mail: fengnq@tsinghua.edu.cn

**Keywords:** concrete, chloride-ion resistant agent, permeability, durability, mortar

## Abstract

The influence of a chloride-ion adsorption agent (Cl agent in short), composed of zeolite, calcium aluminate hydrate and calcium nitrite, on the ingress of chloride ions into concrete and mortar has been experimentally studied. The permeability of concrete was measured, and the chloride ion content in mortar was tested. The experimental results reveal that the Cl agent could adsorb chloride ions effectively, which had penetrated into concrete and mortar. When the Cl agent was used at a dosage of 6% by mass of cementitious materials in mortar, the resistance to the penetration of chloride ions could be improved greatly, which was more pronounced when a combination of the Cl agent and fly ash or slag was employed. Such an effect is not the result of the low permeability of the mortar, but might be a result of the interaction between the Cl agent and the chloride ions penetrated into the mortar. There are two possible mechanisms for the interaction between the Cl agent and chloride ion ingress. One is the reaction between calcium aluminate hydrate in the Cl agent and chloride ions to form Friedel’s salt, and the other one is that calcium aluminate hydrate reacts with calcium nitrite to form AFm during the early-age hydration of mortar and later the NO_2_^−^ in AFm is replaced by chloride ions, which then penetrate into the mortar, also forming Friedel’s salt. More research is needed to confirm the mechanisms.

## 1. Introduction

Since chloride ions constitute one of the main factors causing corrosion of reinforcement, chloride ion penetration in concrete is of great concern [1,2], with regard to durability of reinforced concrete structures subjected to chloride ion attack in coastal areas [3,4] or due to use of deicing salt [5].

A common approach to counter chloride ion attack is to decrease the permeability of concrete, characterized by dense microstructure [6–9], usually at a low water/binder (W/B) ratio and incorporating mineral admixtures. For this purpose, mineral admixtures mainly play the effect of densification of pore-structures in concrete [10], as well as binding chloride ions in concrete [9,11]. The mechanism for chloride binding of mineral admixtures is similar to that of hydrated cement paste. Hydrated cement paste can bind chloride ions both chemically and physically. The chemical binding is due to conversion of hydroxyl AFm to chlorocomplexes typically as Friedel’s salt, 3CaO·Al_2_O_3_·CaCl_2_·10H_2_O, controlled either by an adsorption mechanism where Friedel’s salt forms due to the adsorption of chloride ions in the pore solution of paste into the principal interlayers of the AFm structure, or by an ion exchange mechanism where chloride ions bind with the AFm hydrates to form Friedel’s salt by an ion-exchange with the OH– ions present in the interlayers of the principal layer, [Ca_2_Al(OH^−^)_6_·*n*H_2_O]^+^ [12–14]. The physical binding is a result of van der Waals attraction and electrostatic forces between chloride and cement hydrates mainly as C–S–H gel [13]. Mineral admixtures can release active Al_2_O_3_ to react with Ca(OH)_2_ provided by cement hydration via the Pozzolanic reaction to produce hydroxyl AFm which will further bind chloride chemically [15], meanwhile mineral admixtures can also release active SiO_2_ to produce C–S–H gel which will further bind chloride physically.

The influence of a given mineral admixture on chloride binding of concrete or mortar depends both on the type of mineral admixture and its reactivity. It is of note that silica fume may reduce the chloride binding capacity of concrete, whilst ground granulated blast furnace slag (GGBS) can increase the chloride binding capacity since GGBS promotes formation of more Friedel’s salt [16]. Another research work found that whether or not the addition of fly ash (FA) and GGBS increase chloride uptake depends to a large extent on the reactivity of FA and GGBS related to their composition and fineness, thus the decisive parameter for chloride resistance of concrete is the permeability while the influence of chloride binding is less important when the mineral admixture employed is of relatively low reactivity [17].

Previous research proved that zeolite as a particular type of mineral admixture, different from fly ash (FA) and ground granulated blast furnace slag (GGBS), has both strengthening effect due to its soluble SiO_2_ and Al_2_O_3_ forming C–S–H and C_4_AH_13_ via the Pozzolanic reaction to increase the strength of concrete [2,18], and ion adsorption ability to bind alkali ions such Na^+^ or K^+^ into the porous structure of zeolite [19,20].

In light of the formation of Friedel’s salt to bind chloride ions and of zeolite’s adsorption of alkali ions, it presents a promising way to use a combination of calcium aluminate hydrate and zeolite as a chloride-ion adsorption agent, in which calcium aluminate hydrate can bind chloride ions to penetrate into concrete or mortar and zeolite can absorb alkali ions such Na^+^ or K^+^ from the pore solution to maintain the ionic charge neutrality. Moreover, calcium nitrite *i.e.* Ca(NO_2_)_2_ can also be mixed into the chloride-ion adsorption agent, since calcium nitrite is a commonly used inhibitor for protecting reinforcement in concrete from corrosion induced by chloride attack [21].

This paper presents an experimental investigation on the effect of a chloride-ion adsorption agent (Cl agent in short), composed of zeolite, calcium aluminate hydrate and calcium nitrite, on the chloride ion binding behavior of concrete and mortar. A series of concrete and mortar samples were prepared and tested to identify the characteristics of mechanical strength, permeability, and the ability of the Cl agent to bind chloride ions.

## 2. Results and Discussion

### 2.1. Concrete

#### 2.1.1. Influence of the Cl Agent on Strength of Concrete

The results of compressive strength and tensile splitting strength of concrete are given in Tables 1 and 2, respectively. It can be found that, compared with the compressive strength of the control concrete, slag had the most significant strengthening effect and the Cl agent also had a strengthening effect but second to slag. However, fly ash had a negative effect on compressive strength of concrete. Consistent with the respective strengthening effects of both slag and the Cl agent, the combination of slag and the Cl agent gave the highest compressive strength of concrete, while the combination of fly ash and the Cl agent gave a relatively low compressive strength, as shown in Table 1. The similar effects of slag, the Cl agent, and fly ash, can also be found in the results of the tensile splitting strength in Table 2. These results reveal that the Cl agent has a strengthening effect on concrete, consistent with previous researches [2,18], and such a strengthening effect is more significant when a combination of Cl agent and slag is used.

#### 2.1.2. Influence of the Cl Agent on Permeability of Concrete

**Figure 1.**
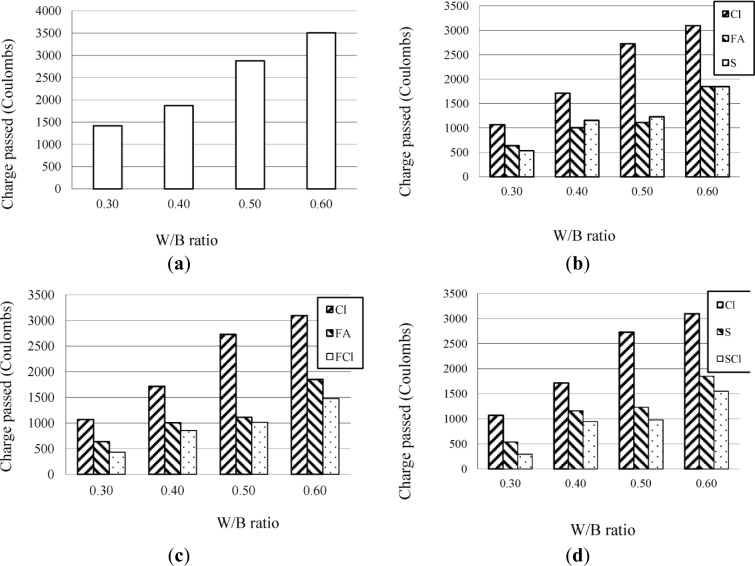
Results of chloride ion penetration through concrete. (**a**) Charge passed of control concrete; (**b**) Charge passed of concrete with one type of mineral admixture; (**c**) Charge passed of concrete with the Cl agent and fly ash; (**d**) Charge passed of concrete with the Cl agent and slag.

The results of the chloride ion penetration tests on a series of concrete at W/B ratios of 0.60, 0.50, 0.40, and 0.30 are given in Figure 1 respectively.

It can be seen that W/B ratio had the most significant effect on the permeability of concrete. Concrete prepared in this investigation at a W/B ratio lower than 0.40 was so dense, that it had a satisfactory permeability and hence chloride ions could not penetrate into it. Furthermore, the type of mineral admixture, especially fly ash or slag at a dosage of 30% by mass, also had a significant effect on the permeability of concrete, which results from the Pozzolanic reaction of mineral admixture [8,9,22]. The Cl agent at a dosage of 6.0% had only a slightly better effect on the permeability of concrete. However, it should be noted that a combination of the Cl agent and mineral admixtures such as fly ash or slag, had a more significant effect on permeability than the Cl agent, fly ash, or slag used alone. Such an effect of combination of the Cl agent and mineral admixtures is very consistent with previous reports [23,24].

### 2.2. Mortar

**Figure 2.**
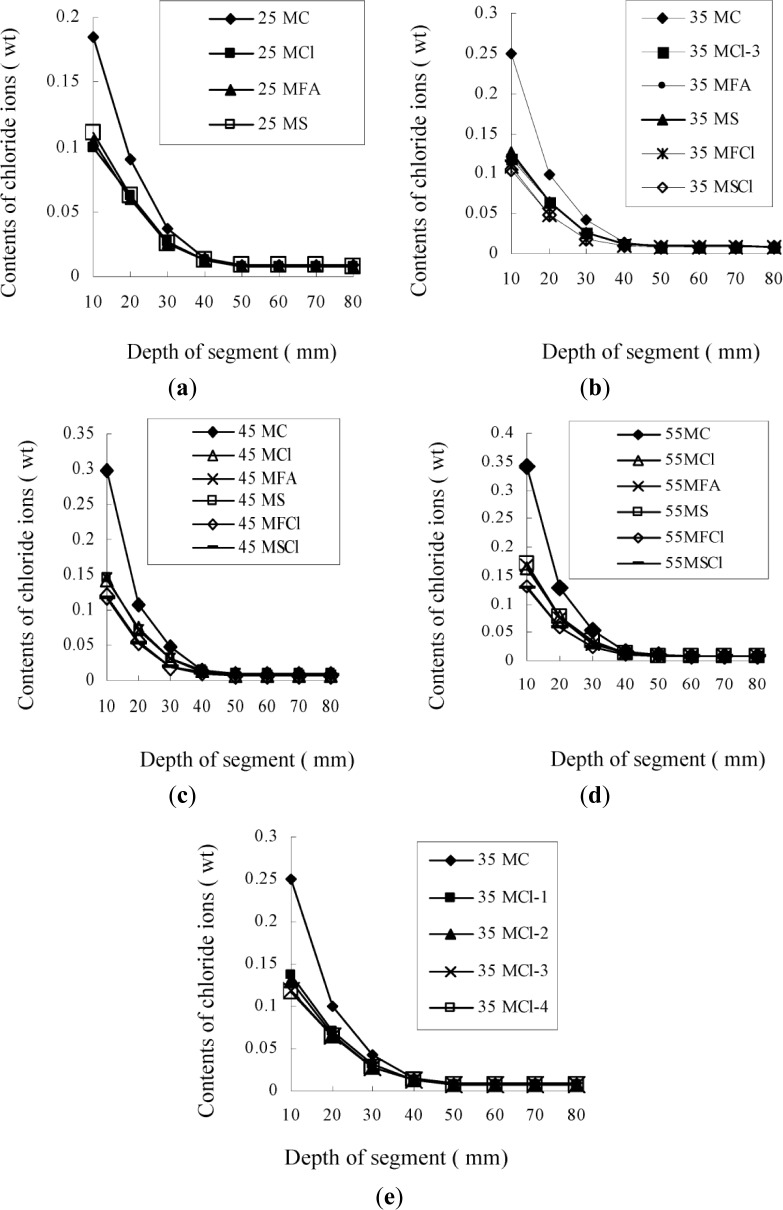
Results of contents of chloride ions in a series of segments of mortar, with a depth of 10–80 mm. (**a**) Mortar at 0.25 W/B ratio; (**b**) Mortar at 0.35 W/B ratio; (**c**) Mortar at 0.45 W/B ratio; (**d**) Mortar at 0.55 W/B ratio; (**e**) Mortar at 0.35 W/B ratio, incorporating the Cl agent at various dosages.

The results of the chloride ion content tests on a series of mortar at W/B ratios of 0.25, 0.35, 0.45, and 0.55 are given in Figure 2. It can be seen from Figure 2 that the Cl agent had a considerable effect on the chloride ion contents detected in mortar. When using the Cl agent at a dosage from 2.0% to 6.0%, the free chloride ion content decreased considerably. Beyond a dosage of 6.0%, there was almost no further decrease in the chloride ion content, so that the maximum dosage of the Cl agent should be 6.0%, when it is used alone. Since the relationship between the free chloride ion content (*C*_f_), the bound chloride ion content (*C*_b_) and the content of total chloride ions (*C*_t_) penetrated into mortar can be presented by Equation (1) [9,25], it is understandable that, for a series of mortar or concrete with relatively similar permeabilities and the same exposure to chloride ions, the less the free chloride ion content measured, the more the chloride ions are bound in the mortar or concrete [15].

$$C_{f}=C_{t}-C_{b}$$ (1)

**Figure 3.**
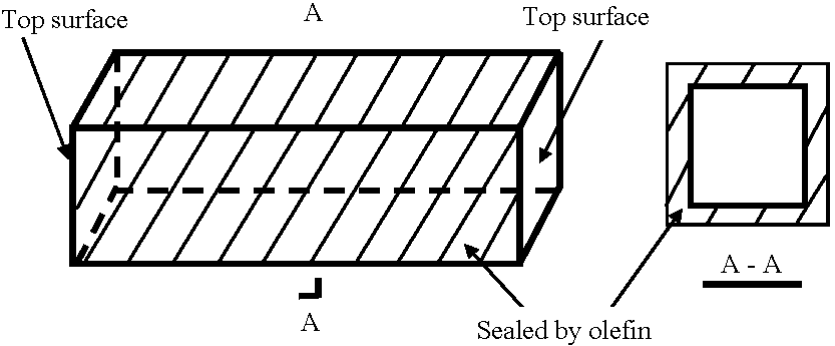
Sealed mortar specimen to be soaked in sodium chloride solution.

Furthermore, it can also be found that using a combination of the Cl agent and mineral admixtures was better than using the Cl agent or mineral admixtures alone, in terms of the chloride ion content tested in mortar. For example, in the segment 0–10 mm of mortar at 0.35 W/B, the chloride ion content was 0.105% when using a combination of the Cl agent at 4.0% and slag at 20% (as 35MSCl in Figure 2b, while it was 0.131% when using the Cl agent at 4.0% (as 35MCl-2 in Figure 2e or 0.128 when using slag at 30% alone (as 35MS in Figure 2b. Although in the latter case, the dosage of slag at 30% was greater than the total dosage of the combination of the Cl agent at 4.0% and slag at 20%, the chloride ion content was higher when using the combination of the Cl agent and slag than that for using slag alone, which might be attributed both to the effect of the Cl agent on the chloride ion content in mortar, and the effect of the chloride agent and slag on the permeability of mortar for enhancing the impermeability of mortar *i.e.*, the resistance to chloride penetration as shown in Figure 3d.

Nevertheless, with regard to the effect of the Cl agent, it is noteworthy that the results of the chloride ion content in mortar were different from those of the chloride ion penetration tests on concrete. As can be seen in Figure 1, the Cl agent at a dosage of 6.0% had only a slightly better effect on the permeability of concrete, the low free chloride contents in mortar incorporating the Cl agent at 6.0%, as shown in Figure 2, were not a result of low permeability of mortar, but a result of interaction between the Cl agent and chloride ions penetrated into the mortar [25–27], which increase the chloride-binding capacity of the concrete or mortar.

There are two possible mechanisms for the interaction between the Cl agent and chloride ion ingress. One is the reaction between calcium aluminate hydrate in the Cl agent and chloride ions to form Friedel’s salt, and the other one may be that calcium aluminate hydrate reacts with calcium nitrite to form AFm during the early-age of hydration of the mortar [28,29] and later the NO_2_^−^ in AFm is replaced by chloride ions, which penetrate into the mortar, to form Friedel’s salt. Parallel to the chloride binding process, alkali ions suchas N^+^ or K^+^ in pore solution can be absorbed by the zeolite of the Cl agent to maintain the ionic charge neutrality.

Obviously, in the present research, the Cl agent increases the chloride-binding capacity of mortar or concrete considerably more than the usual Pozzolanic minerals such as fly ash or slag. Nevertheless, more research is needed to reveal the process of the chemical reaction between the Cl agent, calcium nitrite, and chloride ions.

## 3. Experimental Details

### 3.1. General

Coarse aggregate of crushed limestone with sizes ranging from 5 mm to 25 mm, natural river sand with fineness modulus of 2.5, ordinary Portland cement of 42.5 MPa grade, and mineral admixtures such as fly ash and ground granulate blast-furnace slag were employed. Properties of cement, fly ash and slag are given in Table 3. Naphthalene-based superplasticizer was used to maintain the slump of mixtures around 150 mm. Specimens of a total of 24 types of concrete in the form of 100 mm × 100 mm × 100 mm cubes and ϕ100 × 50 mm cylinders were prepared. The mix proportions of the 24 types of concrete are given in Table 4.

The Cl agent was composed of zeolite, calcium aluminate hydrate and calcium nitrite [30,31]. It was used at a dosage up to 6.0% by mass of the content of cementitious materials in concrete and up to 8.0% of the content of cementitious materials in mortar respectively.

Specimens of a total of 25 types of mortar in the form of 40 mm × 40 mm × 160 mm prisms were prepared. The mix proportions of the 25 types of mortar are given in Table 5, in which cement, sand, mineral admixtures and the Cl agent were the same as those for concrete.

After demolding for one day, all the specimens were cured in water at 20 °C until 28 days, and then cured in air at a temperature of 20 °C and R.H. of 50% (for concrete), or sealed with olefin on four lateral surfaces of each specimen at 28 days as shown in Figure 3 and then soaked in a sodium chloride solution at a concentration of 3.0% by weight until 90 days (for mortar).

### 3.2. Test Methods

Concrete: Permeability was measured on cylinders at 56 days by using the method of ASTM C 1202–2007, entitled “Electrical Indication of Concrete’s Ability to Resist Chloride Ion Penetration” [32]. Determination of compressive strength and tensile splitting strength was conducted on cubes at 28 days, according to the China standard GB/T 50081-2002 which is similar to BS 1881: Part 116.

**Figure 4.**
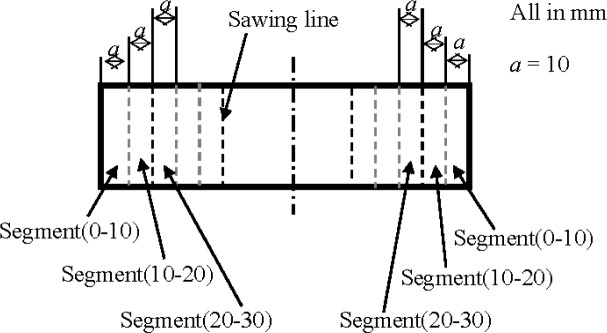
Specimen sawn into segments after soaking in sodium chloride solution over 90 days.

Mortar: After cured in water over 28 days, 40 mm × 40 mm × 160 mm prisms of mortar were sealed with olefin on four lateral surfaces of each specimen, but the other two top surfaces remained unsealed, as shown in Figure 3. After such sealing treatment, the prisms were soaked in a sodium chloride solution at a concentration of 3.0% by weight over 90 days. Then the prisms were taken from the sodium chloride and washed with distilled water. After drying, a prism was sawn into a series of segments at a distance each of 10 mm from the top surface, and the same was done on the other side of the prism, as shown in Figure 4. Each segment after sawing was ground into fine powder. According to the China standard JTJ 270–1998, “Test specifications for concrete in water transport engineering” [33], chemical analysis was conducted on the segments to measure the content of water soluble chloride ions in them, which should be the content of free chloride ion [22]. Two prisms a batch were used to obtain four data for the chloride ion content of a segment with a specific depth, from which an average was obtained in the form of weight percentage of mortar.

## 4. Conclusions

(1) The chloride-ion adsorption agent (Cl Agent), composed of zeolite, calcium aluminate hydrate and calcium nitrite, has a considerable effect on the chloride ion contents detected in mortar. When using the Cl agent at a dosage of 2.0%–6.0%, the free chloride ion content decreased considerably. Such an effect is not a result of the low permeability of mortar, but could be a result of interaction between the Cl agent and the chloride ions penetrated into mortar. It is estimated that the Cl agent increases the chloride-binding capacity of mortar or concrete considerably more than the usual Pozzolanic minerals such as fly ash or slag.
(2) There are two possible mechanisms for the interaction between the Cl agent and chloride ion ingress. One is the reaction between calcium aluminate hydrate in the Cl agent and chloride ions to form Friedel’s salt, and the other one may be that calcium aluminate hydrate reacts with calcium nitrite to form AFm during the early-age hydration of mortar and later the NO_2_^−^ in the AFm is replaced by chloride ions, which penetrate into the mortar, to form Friedel’s salt.
(3) The Cl agent has a strengthening effect on concrete, and such a strengthening effect is more significant when a combination of the Cl agent and slag is used. Furthermore, the combination of the Cl agent and mineral admixtures such as fly ash or slag, has a more significant effect on permeability than the Cl agent, fly ash, or slag used alone.
(4) More research is needed to reveal the process of the chemical reaction between the Cl agent, calcium nitrite, and chloride ions and confirm the mechanisms proposed in this research.

## Figures and Tables

**Table 1.** Compressive strength of concrete at 28 days (mean values and associated errors).

Type of concrete	Mean values and associated errors			
	0.30 (W/B Ratio)	0.40 (W/B Ratio)	0.50 (W/B Ratio)	0.60 (W/B Ratio)
Con	67.4 ± 2.5	54.6 ± 2.3	45.3 ± 1.6	39.3 ± 1.7
Cl	70.5 ± 3.2	54.9 ± 1.9	49.6 ± 2.0	42.3 ± 1.4
FA	64.3 ± 2.1	50.5 ± 2.4	46.7 ± 1.3	37.9 ± 1.7
S	71.2 ± 2.7	57.6 ± 2.5	51.8 ± 1.9	43.1 ± 1.6
FCl	64.0 ± 1.9	49.1 ± 2.2	47.4 ± 1.7	39.8 ± 2.1
SCl	76.1 ± 3.2	59.9 ± 2.7	53.4 ± 2.1	45.5 ± 1.3

**Table 2.** Tensile splitting strength of concrete at 28 days (mean values and associated errors).

Type of concrete	Mean values and associated errors			
	0.30 (W/B Ratio)	0.40 (W/B Ratio)	0.50 (W/B Ratio)	0.60 (W/B Ratio)
Con	5.3 ± 0.2	4.3 ± 0.2	3.8 ± 0.2	3.3 ± 0.3
Cl	5.3 ± 0.3	4.4 ± 0.2	3.9 ± 0.3	3.5 ± 0.2
FA	5.2 ± 0.2	4.1 ± 0.3	3.7 ± 0.1	3.0 ± 0.1
S	5.4 ± 0.1	4.5 ± 0.2	4.0 ± 0.2	3.5 ± 0.2
FCl	5.3 ± 0.3	4.3 ± 0.2	3.9 ± 0.3	3.1 ± 0.2
SCl	5.4 ± 0.2	4.5 ± 0.3	4.1 ± 0.2	3.6 ± 0.2

**Table 3.** Properties of cement, fly ash and slag.

Properties	Cement	Fly ash	Slag
*Chemical composition (% by mass)*			

SiO_2_	21.16	48.58	39.74
Al_2_O_3_	4.17	17.81	10.27
CaO	62.25	15.27	35.38
Fe_2_O_3_	3.92	7.43	1.65
SO_3_	2.59	3.76	1.52
MgO	2.33	1.92	9.83
Loss on ignition	2.56	2.05	0
Na_2_O	0.14	2.28	0.34
K_2_O	0.39	0.86	0.77
Cl^−^	0.009	0	0.012

*Physical properties*			

Specific gravity	3.10	2.25	2.86
Blaine (cm^2^/g)	3845	2562	2654

*Compressive strength (MPa)*			

3 days	24.5	–	–
28 days	52.6	–	–

**Table 4.** Mix proportions and 28-day strength (MPa) of concrete.

Type	W/B	Quantity of raw materials (kg/m^3^)						
		Cement	Sand	Gravel	Water	Cl agent	Fly ash	Slag
30 Con	0.30	550	750	950	165	–	–	–
30 Cl		517	750	950	165	33	–	–
30 FA		385	750	950	165	–	165	–
30 S		385	750	950	165	–	–	165
30 FCl		352	750	950	165	33	165	–
30 SCl		352	750	950	165	33	–	165

40 Con	0.40	450	750	1000	180	–	–	–
40 Cl		423	750	1000	180	27	–	–
40 FA		315	750	1000	180	–	135	–
40 S		315	750	1000	180	–	–	135
40 FCl		288	750	1000	180	27	135	–
40 SCl		288	750	1000	180	27	–	135

50 Con	0.50	400	800	1000	200	–	–	–
50 Cl		376	800	1000	200	24	–	–
50 FA		280	800	1000	200	–	120	–
50 S		280	800	1000	200	–	–	120
50 FCl		256	800	1000	200	24	120	–
50 SCl		256	800	1000	200	24	–	120

60 Con	0.60	350	800	1100	210	–	–	–
60 Cl		329	800	1100	210	21	–	–
60 FA		245	800	1100	210	–	105	–
60 S		245	800	1100	210	–	–	105
60 FCl		224	800	1100	210	21	105	–
60 SCl		224	800	1100	210	21	–	105

**Table 5.** Mix proportions of mortar.

Type	W/B	Relative quantity of raw materials (by mass)					
		Cement	Sand	Water	Cl Agent	Fly ash	Slag
25 MC	0.25	1	2.5	0.25	–	–	–
25 MCl		0.94	2.5	0.25	0.06	–	–
25MFA		0.70	2.5	0.25	–	0.30	–
25 S		0.70	2.5	0.25	–	–	0.30

35 MC	0.35	1	2.5	0.35	–	–	–
35 MCl-1		0.98	2.5	0.35	0.02	–	–
35 MCl-2		0.96	2.5	0.35	0.04	–	–
35 MCl-3		0.94	2.5	0.35	0.06	–	–
35 MCl-4		0.92	2.5	0.35	0.08	–	–
35 MFA		0.70	2.5	0.35	–	0.30	–
35 MS		0.70	2.5	0.35	–	–	0.30
35 MFCl		0.76	2.5	0.35	0.04	0.20	–
35 MSCl		0.76	2.5	0.35	0.04	–	0.20

45 MC	0.45	1	2.5	0.45	–	–	–
45 Cl		0.94	2.5	0.45	0.06	–	–
45 MFA		0.70	2.5	0.45	–	0.30	–
45 MS		0.70	2.5	0.45	–	–	0.30
45 MFCl		0.76	2.5	0.45	0.04	0.20	–
45 MSCl		0.76	2.5	0.45	0.04	–	0.20

55 MC	0.55	1	2.5	0.55	–	–	–
55 MCl		0.94	2.5	0.55	0.06	–	–
55 MFA		0.70	2.5	0.55	–	0.30	–
55 MS		0.70	2.5	0.55	–	–	0.30
55 MFCl		0.76	2.5	0.55	0.04	0.20	–
55 SCl		0.76	2.5	0.55	0.04	–	0.20
